# Inflows of foreign-born physicians and their access to employment and work experiences in health care in Finland: qualitative and quantitative study

**DOI:** 10.1186/1478-4491-12-41

**Published:** 2014-08-07

**Authors:** Hannamaria Kuusio, Riikka Lämsä, Anna-Mari Aalto, Kristiina Manderbacka, Ilmo Keskimäki, Marko Elovainio

**Affiliations:** 1 National Institute for Health and Welfare (THL), Service System Research Unit, Mannerheimintie 103 b, P.O. Box 30, FI-00271 Helsinki, Finland; 2 National Institute for Health and Welfare (THL), Health and Social Services, Mannerheimintie 103 b, P.O. Box 30, FI-00271 Helsinki, Finland; 3 School of Health Sciences, University of Tampere, Mannerheimintie 103 b, P.O. Box 30, FI-00271 Helsinki, Finland

**Keywords:** General practitioners, Finland, Physicians, Primary care, Professional migration

## Abstract

**Background:**

In many developed countries, including Finland, health care authorities customarily consider the international mobility of physicians as a means for addressing the shortage of general practitioners (GPs). This study i) examined, based on register information, the numbers of foreign-born physicians migrating to Finland and their employment sector, ii) examined, based on qualitative interviews, the foreign-born GPs’ experiences of accessing employment and work in primary care in Finland, and iii) compared experiences based on a survey of the psychosocial work environment among foreign-born physicians working in different health sectors (primary care, hospitals and private sectors).

**Methods:**

Three different data sets were used: registers, theme interviews among foreign-born GPs (n = 12), and a survey for all (n = 1,292; response rate 42%) foreign-born physicians living in Finland. Methods used in the analyses were qualitative content analysis, analysis of covariance, and logistic regression analysis.

**Results:**

The number of foreign-born physicians has increased dramatically in Finland since the year 2000. In 2000, a total of 980 foreign-born physicians held a Finnish licence and lived in Finland, accounting for less than 4% of the total number of practising physicians. In 2009, their proportion of all physicians was 8%, and a total of 1,750 foreign-born practising physicians held a Finnish licence and lived in Finland. Non-EU/EEA physicians experienced the difficult licensing process as the main obstacle to accessing work as a physician. Most licensed foreign-born physicians worked in specialist care. Half of the foreign-born GPs could be classified as having an ‘active’ job profile (high job demands and high levels of job control combined) according to Karasek’s demand-control model. In qualitative interviews, work in the Finnish primary health centres was described as multifaceted and challenging, but also stressful.

**Conclusions:**

Primary care may not be able in the long run to attract a sufficient number of foreign-born GPs to alleviate Finland’s GP shortage, although speeding up the licensing process may bring in more foreign-born physicians to work, at least temporarily, in primary care. For physicians to be retained as active GPs there needs to be improvement in the psychosocial work environment within primary care.

## Background

An increasing shortage of general practitioners (GPs) threatens the effective functioning of primary health care in many countries. In the USA, more than 30% of all rural counties have a shortage of GPs [1]. Australia, too, suffers from a GP shortage in rural areas and increasingly also in metropolitan areas [2,3]. In Finland, health care is mainly publicly funded, and responsibility for running the health care system is delegated to local government. Public primary health care is provided by district health centres; local authorities have their own health centres or form joint municipal boards. Primary health care services provided by the private health care sector account for 16% of outpatient physician visits. In addition, occupational health services account for about 13% of outpatient physician visits; these are mainly provided by private sector firms [4]. In Finland, the shortfall from the required number of physicians at primary care health centres was 6% in 2010 [5], considering both domestic and foreign-born physicians.

However, of the total number of GPs, 18% were substitutes and nearly 6% hired from labour-leasing companies [5]. Foreign-born physicians fill the gap in the medical workforce in many developed countries. For example, in the USA, Australia, and Canada in 2004, nearly 25% of practising physicians were foreign-born [6]. International mobility of physicians to Finland has been low, although the inflow has increased since the end of 1990s [7]. In 2010, Finland had 1,297 foreign-born physicians licensed to practise, about 10% of the total GP workforce.

More attractive alternatives, such as better working conditions or pay, have been shown to motivate physicians to seek employment abroad [8]. Opportunities for employment are influenced by health and migration policies and the health care system of the receiving country. Physicians may practise their profession only if their qualifications are recognised, if they obtain a residence permit, and if there are jobs available. Even where qualifications are recognised, foreign-born physicians may face a language barrier and lack of knowledge of clinical procedures and the wider organisational culture (e.g., [9]).

To settle in Finland, EU/EEA citizens must have a residence permit issued by the police, while non-EU/EEA citizens must have a residence permit issued by the Finnish Immigration Service [10]. Moreover, physicians must be licensed by the National Supervisory Authority for Welfare and Health (Valvira) in order to be allowed to practise in Finland. Within the European Union, the qualifications of physicians trained in the EU/EEA are recognised according to an EU Directive [11], but there are no such standard procedures within the EU for physicians trained outside the EU/EEA. According to the Directive, recognition of the professional qualification may be granted without consideration of the appropriate language skills. In Finland, however, employers are required to ensure that their employees have sufficient proof of language skills. Physicians whose qualifications were obtained outside the EU/EEA have to produce evidence of sufficient language skills (e.g., qualifications awarded by a recognised language institution), complete additional studies, and/or pass an examination in Finnish in order to be licensed to practice their profession. The examination consists of three parts covering basic knowledge of clinical medicine and health care, basic knowledge of the health-care system in Finland (including issues central to the practice of medicine in Finland), and clinical skills.

There is some evidence from the USA that foreign-born physicians are more likely to practise in underserved areas and in primary health care than native physicians [12]. However, the opposite is demonstrated by the findings of Baer et al. [13]. An earlier study conducted in the USA found that foreign-born GPs were less satisfied with primary health care work than native GPs [14]. A previous Finnish study found that the intent to leave a job is more prevalent among foreign-born GPs than Finnish GPs [15]. It has repeatedly been shown that the psychosocial work environment, pertaining to interpersonal and social interactions in the workplace, plays a central role in the work-related wellbeing and job satisfaction of employees. According to Karasek’s [16] widely used demand-control model, a job with low control possibilities combined with high demands, such as time pressure, is particularly distressing, while a high-stress job has been shown to have negative impacts for both individual employees and the organisation [17,18]. Linzer found that time pressure and chaotic workplace environments, low work control, and an unfavourable organisational culture were associated among GPs with physician dissatisfaction, stress, burnout, and intent to leave a job [19]. A previous study in Finland showed GPs to be less committed to their job than other physicians because of poorer working conditions in primary health care [20]. A previous follow-up study in Finland showed that patient-related stress and frustration with electronic patient record systems increased in Finland between 2006 and 2010 [21]. Another earlier Finnish study suggested that public-sector physicians were less satisfied and committed to their job than private-sector physicians. Private sector physicians also described fewer psychosocial disorders and sleep problems [22]. In Sweden, too, public-sector physicians seemed less satisfied with their workplace environment than private-sector physicians [23]. However, little is known about the experiences of foreign-born physicians in a receiving country with regard to the psychosocial workplace environment or job satisfaction and about whether there are differences in these between the various health care sectors. The aim of the present study was, thus, i) to examine, based on register information, the numbers of foreign-born physicians migrating to Finland and their employment sector; ii) to examine, based on qualitative interviews, the foreign-born GPs’ experiences of accessing employment and work in primary health care in Finland; and, finally, iii) to compare experiences of the psychosocial workplace environment among foreign-born physicians working in various health care sectors (primary health care, hospitals, and private sector). Three data sources were used in order to establish a comprehensive picture of foreign-born physicians’ migration, access to employment, and work experiences in Finland. The term ‘foreign-born physician’ refers here to a physician born and trained outside Finland, whether a foreign national or a person born abroad who now holds Finnish citizenship. The definition does not include physicians who were born in Finland but trained abroad.

## Methods

### Data

Three different data sets were used for this study to answer the study question above. The study questions are linked to the data sources so that the register data answers the first study question, qualitative data answers the second, and the survey questionnaire answers the third.

#### Study question 1

The number of foreign-born physicians migrating to Finland and their employment sector were obtained from administrative registers such as those of the tax administration and form the basis of population censuses maintained by Statistics Finland. The data obtained from Statistics Finland came from the publicly available databases on their website ( http://www.tilastokeskus.fi/). The number of new licences for foreign-born physicians was obtained from the National Supervisory Authority for Welfare and Health (Valvira). The contact information for foreign-born physicians was applied for and obtained from Valvira for the purpose of conducting this study. The numbers of foreign-born physicians reported in the results section were calculated from the data obtained.

#### Study question 2

Qualitative data was collected from foreign-born GPs working in Finland for the purpose to document their experiences of the licensing process and employment and work in primary care. Qualitative findings were also partly used to design measures in a questionnaire survey. The absence of previous research on this issue in Finland led us to choose theme interviews as a research method, and thereby to aim to form hypotheses of the potential problems encountered by foreign-born GPs in employment and working life in Finland. The 12 interviews provided us with enough information on the topics of interest. The interviews were carried out at six health centres between September 2009 and January 2010. The themes of the interviews were related to experiences concerning how the GPs came to Finland, their experiences of the licensing process and integration into the Finnish health care system, job satisfaction, language skills, and their career choices and future plans. The interviews lasted from 45 to 90 minutes, and were audio-recorded with the interviewees’ permission and transcribed verbatim. The transcript of the tapes consisted of 106 pages of single-spaced text. The interviewees, of whom seven were women, varied in age from 30 to 60. The range of the length of their stay in Finland was 4 to 19 years. Six originally came from Russia, two from EU/EEA Member States, and the remaining four from countries outside these areas.

#### Study question 3

Once interviews were complete, we conducted a web-based questionnaire survey to examine foreign-born physicians’ experiences of the psychosocial work environment and job satisfaction. Although the measures in the survey partly arose from the interviews, we also included validated measures from previous studies among native physicians. The invitation went to all foreign-born physicians licensed to practise and living in Finland in 2010 (n = 1,297). This number is smaller compared to register based information due to stricter selection rules: we included only those physicians who have been licenced and live in Finland. While intended for answering in Finnish, the questionnaire was also translated into English, Swedish, Russian, and Estonian. A link to the electronic questionnaire was sent to physicians during autumn 2010 by email, with up to three reminders. After the first round, printed questionnaires were mailed to non-responders with one reminder. Altogether 553 foreign-born physicians responded out of the original 1,297, giving a response rate of 42%. For the present analysis the sample was restricted to those working in the health care sector (public primary or specialized care or private sector, n = 498).

Ethical approval for the study was obtained from the Ethics Committee of the National Institute of Health and Welfare (Approval number 7/2010).

### Measures

High job demand was measured by a 5-item scale (e.g., ‘Constant rush and pressure due to non-completed work’, response scale 1 = never, 2 = seldom, 3 = occasionally, 4 = quite often, 5 = all the time) derived from Harris’ (1989) stress index [24] (α = 0.87). Job control was measured by decision authority scale (3 items, α = 0.76) derived from Karasek’s Job Content Questionnaire [25]. An item example is ‘I can make independent decisions in my work’, with response alternatives 1 = completely disagree, 2 = somewhat disagree, 3 = undecided, 4 = somewhat agree, 5 = strongly agree. For descriptive purposes, job demand and job control scales were categorized so that scores 3 or lower meant low job demand/control and scores above 3 high job demand/control. From these categorized variables we computed a combined variable to represent Karasek’s demand-control model: passive work (1 = low job demand and low control, 0 = other combinations), active work (1 = high job demand and high control, 0 = other combinations), high strain work (1 = high job demand and low control, 0 = other combinations), and low strain work (1 = low job demand and high control, 0 = other combinations).

Patient-related stress was measured with a 3-item scale (α = 0.84) derived from the health care stress questionnaire [26] (item example: ‘Patients are unwilling to co-operate and are passive’). Stress related to patient information systems was measured with a self-developed 2-item scale (α = 0.82), the two items being ‘constantly changing data-systems’ and ‘poorly working tele-informatic programmes’. Lack of professional support was also measured with a self-developed 2-item scale (item example: ‘possibility to consult’, α = 0.62). Stress related to teamwork was measured with a 4-item scale (e.g., ‘Human relationship problems in the workplace’, α = 0.85) derived from Harris’ stress index [24]. These stress scales used the same response scale as for job demands, and were also categorized using score 3 as a cut-off point.

Job satisfaction was measured by 3 items from Hackman and Oldham’s (1975) Job Diagnostic Survey [27] (e.g., ‘I am satisfied with my work’, α = 0.79). Job involvement was measured by 3 items developed by Lawler and Hall (1970) [28] (e.g., ‘The most important things that happen to me involve my job’, α =0.83). Team climate was measured by a 4-item Team Climate Inventory [29], an item example being ‘We have a "we are together" attitude’ (α =0.88). The response scale in job satisfaction, job involvement, and team climate scales was 1 = completely disagree, 2 = somewhat disagree, 3 = undecided, 4 = somewhat agree, 5 = strongly agree. For descriptive purposes, the variables were categorized using score 3 as cut-off (scores above 3 indicating strong job satisfaction/job involvement and good team climate).

### Analysis

Frequency tables were calculated for trends in migration from Statistics Finland in the years 1990, 1995, 2000, 2005, and 2009. The total numbers of foreign-born physicians were compared to total numbers of Finnish physicians in order to calculate a proportion of foreign-born physicians. The numbers of new licenses were obtained from the National Supervisory Authority for Welfare and Health (Valvira). In addition, foreign-born physicians’ country of origin was obtained from the Medical Association database in the year 2013.

We chose qualitative content analysis as the analysis method for the theme interviews. The analysis proceeded inductively from smaller categories to major categories. The data were coded using Atlas.ti software. After several readings, the data were classified independently by three of the authors (HK, RL, KM) into 81 sub-categories. The results were compared, discrepancies discussed, and data merged into seven categories representing different aspects of the licensing process for foreign-born physicians and their experiences in primary care work.

We analyzed the differences in psychosocial work environment between foreign-born GPs and other foreign-born physicians using ANCOVA in continuous variables adjusting for background factors (age and gender, country of origin, length of stay in Finland, reason for migration and specialization), and we present means and *F*-statistics for these differences. Bonferroni correction was applied in multiple pair-wise comparisons, while in categorical variables (Karasek’s job strain variable) unadjusted differences between GPs and other physicians were first tested by χ^2^ test. We also used multivariate logistic regression analyses to examine the differences between foreign GPs and other foreign physicians, adjusting for background variables. All statistical analyses were conducted using the SPSS software, version 19.0.

### Results from the registers

**Figure 1:**
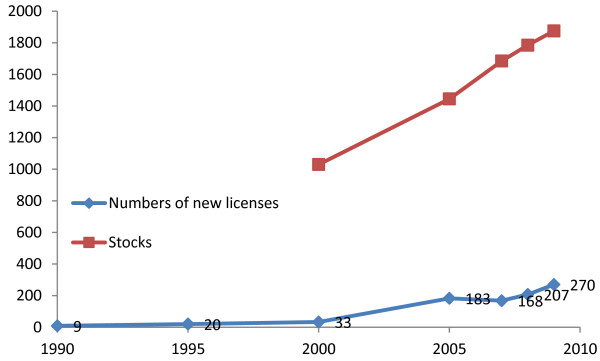
**Numbers of foreign-born physicians’ new licences and stocks in Finland.** Years 1990, 1995, 2000, 2005, and 2009

The number of foreign-born physicians in Finland remained low until the end of the 1990s, but has increased significantly over the last 10 years. According to Statistics Finland, in the year 2000 a total of 980 foreign-born physicians held a Finnish licence and lived in Finland, accounting for less than 4% of the total number of practising physicians. In 2009, their proportion was 8%, and a total of 1,750 foreign-born practicing physicians held a Finnish license and lived in Finland (Figure 1).According to Valvira statistics in 1990, nine foreign-born physicians were given a license to practice in the medical profession in Finland. In 2009, the license was given to 270 foreign-born physicians (Figure 1), compared to the 570 new licences given to Finnish-trained physicians in the same year. Nowadays, the Russian Federation is the most important source country for foreign-born physicians, accounting for around 70 to 80 new licenses per year between 2004 and 2008. According to the Medical Association database in the year 2013, the majority of foreign-born physicians have come from the Russian Federation and Estonia. In 2013, a total of 357 Russian-born physicians were licenced and lived in Finland, and of them 212 had a Finnish nationality. From the Estonian-born, a total of 287 physicians were licenced and lived in Finland, and of these 40 had a Finnish nationality.

In 2009, most of the practicing foreign-born physicians (82%) were employed in health and social services. Public hospitals were the biggest employer (47% of the physicians working in health and social services), while public primary care employed 22% and the private sector employed 9% of the foreign-born physicians. Compared to this, 82% of Finnish-born physicians were employed in health and social services, and of those 43% worked in public hospitals while primary care employed 18%. Finally, the private sector employed 16% of Finnish-born physicians.

### Interview results

One of the major concerns, especially among physicians trained outside the EU/EEA trying to enter the profession in Finland, was access to work in the health care sector made problematic by a difficult licensing process. We identified four themes of problems: lack of information, bureaucratic difficulties, lack of support with language studies, and unfair test requirements. The lack of information was from official sources, such as employment offices or their superiors, about language courses, the licensing process, and jobs available. This was especially true among physicians trained outside the EU/EEA. "*I’ve found that chief physicians, they don’t know how it* [the licensing process] *works for foreign physicians, they don’t know the difference between qualifying in Europe or outside Europe*" (P1). It appears that the Finnish system failed to support the foreign GPs’ language training, and their lack of language skills prevented them from entering the Finnish system. The Finnish language is difficult to learn, and language courses were described as being in short supply or poor quality. "*The students were at very different levels, some did not even know the alphabet, so we spent time just learning letters*" (P10). GPs from outside the EU/EEA described the test requirements for obtaining a licence as excessive, and test requirements or practices were not even consistent over time. "*I remember the hall was so full, about 100 people in there, and three months later they announced that only one person had passed the test. What kind of a test is it that an entire hall full of people can fail it? It’s just not fair*" (P12). Even though foreign GPs were struggling to make decisions based on fragmented information, they had also come up with various strategies for gaining experience in order to achieve their goal of practising medicine in Finland. "*Then I went to Meilahti Hospital and said, I’m a doctor, I want to get to know the system, how it works, I don’t have a licence yet but could come here to learn*" (P3).

Once the foreign-born physicians were licensed and employed they seemed relatively satisfied at work. We identified four themes that describe work in primary health care. The heavy workload seemed to be a common concern and a ‘chronic condition’ among foreign-born GPs. The other themes were mixed; we described them as ‘the role of the orientation to work’ and ‘patient work is satisfying yet stressful’.

Comprehensive work orientation and consultation opportunities were highly valued and often, but not always, experienced at the workplace*.* Foreign physicians were sometimes treated like doctors doing specialist training in general practice, which was much appreciated because they were assigned fewer patients per day, and they also had a senior GP as a support person. Peer support from other foreign physicians was also considered valuable by GPs of foreign origin, especially those who had little working experience: "*There might be a question about treatment where he* [a foreign colleague] *might have more experience of how they do things at the health centre*" (P4). GPs felt that they had good social relationships with colleagues and other team members at the workplace. However, it was also suggested that contacts with colleagues were superficial, and that the Finnish way of communicating could be strange for a foreigner.

Positive feedback from patients increased the GPs’ job-satisfaction and encouraged them in their work. Negative feedback from the patients naturally bothered the GPs, even outside work. The GPs valued no-nonsense patients who were willing to cooperate with the physician*.* Certain patient groups were found to be challenging, e.g., patients with multiple diseases, and their treatment would have required more time than allocated. "*Chronic diseases are the tough ones, there are elderly people who have a lot of different medications and complaints, and the appointment time is limited, and in that time you should be able to find out exactly what the problem is*" (P12). Consumer-oriented patients who had specific expectations and directions for the GP consultation or just wanting a referral to a specialist were found to be demanding and not always appreciative of the work and expertise of the GP.

The heavy workload resulting from the shortage of GPs was considered ‘a chronic condition’ at health centres. Foreign-born GPs worked long hours and sometimes continued working after the health centre was closed. This could be down to a sense of duty, or an inability to cover the workload and paperwork in the time allocated. "*It just wasn’t possible. Everything else* [but patient work] *had to be done in the evening, sometimes until 19.00, they were really long days*" (P3). The foreign-born GPs viewed the general GP shortage as the cause of increased workload and an important negative factor for job satisfaction. "*Physicians may leave for whatever reason, or a position remains unfilled, and the work is just piled on to all the others. We’re busy all the time and people are waiting, patients become impatient and angry, they’re given a 20-minute appointment and they have a hundred and one things to sort out. I’ve never treated them before, and in that time I can’t solve all their problems, but since I do want to help them, the appointment drags into overtime* […]" (P9). GPs maintained that too much time was spent on paperwork, e.g., the time used for making entries in the electronic patient record system, or on administrative meetings during on-call duties. They also found patient information systems to be complex because of the many different systems used, depending on the health-care centres in which they worked.

### Survey results

Table 1 shows the characteristics of the survey study sample. Most foreign-born physicians were female, and the mean age was 44.6 years (range 24–69 years and SD = 10.6). Most of the foreign-born GPs came from the Russian Federation, while other physicians, such as those working in hospitals or in private sectors, came from Estonia or other countries. Most foreign-born GPs and physicians in the private sector reported having obtained their residence permit in Finland for family reasons. The most common reason among physicians in specialized care was work-based migration.

**Table 1:** Characteristics of the survey sample

	**Primary care**	**Specialized care**	**Private sector**			*
**Age** (Mean, (SD)	43 (10)	43 (11)	50 (8)			*F* = 7.3, *P* <0.001
**Women (%)**	54	77	45			χ^2^ = 32.6, *P* <0.001
**Country of origin (%)**				%	n	χ^2^ = 15.7, *P* = 0.003
Estonian	27	35	33	32	150	
Russian	42	25	24	32	151	
Other	31	39	44	37	173	
**Residence permit based on (%)**						χ^2^ = 36.9, *P* <0.001
Ethnic returnee	18	7	10	12	52	
Family	44	32	51	38	168	
Work	35	60	32	48	209	
Refugee/asylum seeker	3	1	7	3	11	
**Arrived in Finland (%)**						χ^2^ = 48.3, *P* <0.001
Before 1989	13	12	40	15	72	
1990–1999	34	29	51	33	155	
2000–2005	30	40	4	33	155	
2006–2010	23	20	4	20	93	
**Specialization (%)**						χ^2^ = 113.6, *P* <0.001
No	45	7	20	22	107	
Ongoing specialization	25	37	14	30	149	
Specialized	30	58	66	48	240	

*Based on χ^2^ test for categorical variables and on F-statistics on continuous variables.

Foreign-born public physicians (GPs and medical specialists) experienced higher job demand in comparison to foreign-born private physicians (Table 2). Private physicians experienced higher job control in comparison to medical specialists. Foreign-born GPs’ experienced more patient-related stress in comparison to foreign-born medical specialists and private physicians. Foreign-born public sector physicians’ frustration with electronic patient record systems and stress related to team work was higher in comparison to private physicians. Job satisfaction differed significantly between foreign-born GPs and foreign-born private physicians. No significant results were found in respect of professional support, job involvement, and team climate among foreign-born GPs, medical specialists, and private physicians.

**Table 2:** Psychosocial work environment among foreign-born physicians according to health care sector

	**Primary care**^**1**^	**Specialized care**^**1**^	**Private sector**^**1**^	***F***	***P***
Job demand	3.39^c^	3.25^c^	1.88^a,b^	29.8	<0.001
Job control	3.97	3.75^c^	4.31^b^	4.9	0.008
Lack of professional support	2.47	2.19	2.24	2.2	0.116
Stress related to information systems	2.98^c^	3.02^c^	1.57^a,b^	17.1	<0.001
Patient related stress	2.87^b,c^	2.51^a^	2.17^a^	8.6	<0.001
Stress related to team work	2.01^c^	2.06^c^	1.16^a,b^	9.1	<0.001
Job satisfaction	3.84^c^	4.09	4.54^a^	5.9	0.003
Job involvement	3.73	3.86	4.06	1.7	0.183
Team climate	3.97	3.93	4.11	0.4	0.659

^1^Age, sex, country of origin, year of migration, permit of residence, specialization;

^a^Differs significantly (*P* ≤0.05) from those working in primary care;

^b^Differs significantly (*P* ≤0.05) from those working in specialized care;

^c^Differs significantly (*P* ≤0.05) from those working in private sector.

In the combined measure for job strain typology [16], half of the GPs were classified as having active work, one third as having low strain work, 16% as having high strain work, and 2% as having passive work (Table 3). GPs differed significantly from physicians working in specialized care or in the private sector in more often having active work, and less often low strain (Table 3). Furthermore, 16% of GPs and 17% of physicians in specialized care had high strain work, while none of the physicians in the private sector was classified in the high strain group. In logistic regression analyses (adjusted for background factors) the differences between GPs and other physicians in active work remained significant (*P*<0.001), as did differences between GPs and physicians in the private sector in low strain jobs (*P*<0.001). The small number of cases in the cells meant that adjustment was not possible in the models for passive and high strain work.

**Table 3:** Type of work according to Karasek’s demand-control model among foreign-born physicians

	**Primary care**	**Specialized care**	**Private sector**		
	**% (n)**	**% (n)**	**% (n)**	**χ**^**2**^	***P***
Passive work	3 (5)	6 (16)	2 (1)	4.1	0.126
Active work	52 (99)	38 (96)	20 (10)	20.3	<0.001
Low strain work	29 (55)	39 (100)	78 (39)	39.6	<0.001
High strain work	16 (30)	17 (43)	0 (0)	9.7	0.008
TOTAL	100 (189)	100 (255)	100 (50)		

Means adjusted for confounders and *F*-statistics.

## Discussion

This study examined how foreign-born GPs entered the profession and how they experienced working in Finnish health centres. Three different data sets were used: register information, theme interviews, and survey data. The study showed that the numbers of foreign-born physicians have increased dramatically in Finland since the year 2000. The shortage of physicians has often been seen as a powerful spur for the international migration of physicians [30]. The increasing inflow of physicians in the Finnish context was partly allowed by Finland joining the EU in 1995. A change in the Finnish policy environment – from a mainly humanitarian-based immigration policy to one of enhancing work-related immigration [31] in response to the challenges of an ageing population and workforce shortages – may also have contributed to the inflow increase. Most foreign-born physicians immigrate to Finland from the Russian Federation or Estonia. Russian physicians appear to be drawn to Finland by kinship ties and family already living there, while Estonian physicians arrive mainly on account of work-related factors. The importance of ‘family proximity’ has demonstrated also among overseas-trained physicians in Australia [32] and geographical proximity in some European countries [33]. Our results support previous findings also from the USA that proximity of the destination country and GDP per capita are the two main predictors of physicians’ migration [34]. Higher salaries and better working conditions have been the main emigration factors for Estonian physicians [35] and for physicians in some other countries [36]. Increasing mobility from Estonia to Finland may have negative impact on health system performance in Estonia, however, we lack information of the size of such a phenomenon. It seems that Finland fails to attract large numbers of physicians from other European countries or overseas probably due to the geographical location and language difficulties.

The slow licensing process for non-EU/EEA physicians hindered access to work in Finland, and was experienced as an uninviting, inconsistent, and confusing process with many obstacles. Key issues were insufficient information on the licensing process, lack of support with language studies, and test requirements viewed as difficult. Similar difficulties have been experienced in Canada with licensing of physicians trained abroad [37,38]. Within Europe, the qualifications of physicians trained in the EU/EEA are recognised by EU Directive [11], but there are no consistent practices for physicians trained outside the EU/EEA [39]. According to the present study, the challenge in the licensing process lies in increasing the availability of information concerning the test requirements, and providing language courses especially for physicians trained outside the EU/EEA. According to Haukilahti, only one out of five non-EU/EEA physicians who took the licensing examination in Finland between 1994 and 2009 passed the examination on their first attempt [40]. Thus, this may indicate that many foreign-born physicians work in areas other than the health care sector or are unemployed.

While most foreign-born physicians worked in specialist care, the sector is large in Finland and the biggest employer of the medical workforce regardless of origin. According to a physician survey from 2009, almost half of the working-age physicians worked in specialized health care and 21% in health centres [41]. This may indicate that foreign-born physicians prefer applying to specialties popular also among Finnish physicians. However, there may be characteristics in hospital work that can make specialized care particularly attractive to foreign-born physicians. The language requirement among some hospital physicians may be less strict compared to GPs because hospitals have several specialties (e.g., surgery or anaesthesiology) which may not require comprehensive language skills. Foreign born physicians may also prefer hospitals because of previous working experience in their country of origin, an ambition to become a specialist, or poorer career options and working conditions in primary care. On the other hand this may also suggest that primary care might not be able to attract foreign-born GPs and, thus, there is a need to consider other options to make work in primary care attractive for foreign-born physicians.

The psychosocial working environment of foreign-born GPs was mainly characterized by high job demand and broad opportunities for controlling their own work. In previous studies, primary care work has been discussed in terms of high strain work or its components – high work load and low job control – which have been associated with poor well-being [14], high absenteeism [15], low commitment [18], and retirement intentions [42]. However, in the present study, half of the foreign-born GPs in the survey could be classified as having an ‘active’ job profile according to Karasek’s demand-control model [22]. Active work was even more common among GPs than among other foreign-born physicians. This kind of work is associated with positive outcomes such as job challenge and satisfaction [43]. The divergent findings could be a result of methodological differences. Usually the demand-control model has been used for predictive purposes, and high vs. low conditions in demand and control have been defined based on distribution of responses in specific data sets. In this study, we used Karasek’s typology for descriptive purposes, and therefore defined high time-pressure and job control based on initial response alternatives of scales. An alternative explanation could be that foreign-born physicians assess their working environment by different standards from native physicians, for example comparing their experiences to those from their country of origin.

Our results from the theme interviews mirrored the survey results in that, on top of the recurrent theme of job demand, the interviews also revealed that multifaceted work in health centres – and the diversity of expertise it requires – may also be experienced as inspiring by some foreign-born physicians. The finding is in line with earlier interview study among Finnish GPs, where the comprehensive work in primary care was experienced as both stressful and positively challenging [44]. Information systems in health care have been severely criticized by physicians [45], and poorly functioning patient electronic systems (ITC) systems were also a major source of stress among foreign-born physicians in the present study, particularly in public primary or specialized care. The interviewees described the ITC systems as complicated to use, with entries taking a long time.

Patient-related stress was more common among foreign-born GPs than among other foreign-born physicians working in other health care sectors. This was also found in previous studies among native physicians [46] and native GPs. The foreign-born GPs interviewed experienced particular challenges with patients with multiple conditions or mental health problems, and with demanding, consumer oriented patients.

Another theme recurring in the interviews was the importance of social relationships and support from colleagues, and foreign-born physicians evaluated team climate positively in the survey, regardless of the health care sector in which they worked. The need for support among foreign-born physicians has also been recognized elsewhere, and orientation and mentoring programmes for foreign-born physicians [47,48] have been found useful in promoting their integration.

This study used three different data sets to look at the same phenomenon from different perspectives, and this may increase the credibility of our results. The qualitative data were based on a relatively small number of interviews. Simultaneously, with survey data, we were able to compare the themes and issues that emerged from the interviews to responses to a survey among foreign-born physicians working in Finnish health care. The results are specific to the Finnish health care context and cannot be generalised directly to other countries. In addition, qualitative data was gathered in the Helsinki metropolitan area, thus the results may not be comparable with foreign-born physicians living in rural areas. Moreover, although six interviewed physicians came from Russian, which is the largest source country of foreign-born physicians in Finland, the remaining interviewed physicians do not reflect the whole range of nationalities of total population of foreign-born GPs in Finland.

In this study, we have only been able to catch the licensed foreign-born physicians who live in Finland, and thus lack information on temporary workers and on physicians who live in Finland but have not been licensed there. Our results regarding the licensing process, therefore, reflect only the experiences of those who had passed through the process or were still involved with it. The interviews were conducted in Finnish, which was not the interviewees’ native language. Although the respondents in the interviews spoke good Finnish, some personal experiences may be easier to discuss in one’s native language. The respondents were able to choose from several language versions of the survey. The response rate among foreign-born physicians was relatively low (42%) and employed and female foreign-born physicians were over represented among foreign-born physicians [49]. Furthermore, the cross-sectional study design of the survey prevents us from making causal interpretations, while results may be over-inflated through the use of self-reported data. To minimize problems with self-reports, we have used well-known validated measures that have shown good reliability.

## Conclusions

While the number of foreign-born physicians has increased rapidly since the year 2000 in Finland, the licensing process was experienced to be exclusive and particularly unfair by foreign-born physicians trained outside of EU/EEA. Most foreign-born physicians seemed to work in specialized medical care which was followed by primary care. Those working in primary care seemed to experience similar problems, such as high job demand and high patient related stress, as reported by native Finnish GPs in previous studies. However, demands were often associated with high job control among foreign-born GP’s and comprehensive work in health centres was found both stressful and inspiring. Improvements are necessary in employee retention and management of the primary care function if we are to keep physicians working as GPs, whether foreign-born or native.

Negative impacts of job demands could be decreased, for example, by dividing tasks between nurses and GPs. This study also suggests that investing in professional support in more detail could make the job of GPs easier. In addition, the ITC system could be unified and make them easier in everyday use. Effective solutions are often context-related, and thus priority should be given to the local and organizational level.

This study indicates that primary care may not be able in the long run to attract a sufficient number of foreign-born GPs to alleviate Finland’s GP shortage. One way to ease foreign-born physicians’ employment in Finland is to speed up the licensing process for foreign-born physicians, e.g., by providing easier access to training and language courses. This could be implemented in workplaces such as health care centres. Speeding up the licensing process may bring in more foreign-born physicians to work, at least temporarily, in primary care.

## Competing interests

HK, RL, KM declare that they have no competing interests. A-MA: I have the following competing interests: I have received funding from the Finnish Work Environment Fund for the study but the Finnish Work Environment Fund had no involvement in its design, data collection, findings, or decision to publish. IK: I have the following competing interests: I am asked to advise the Finnish Ministry of Health and Social Affairs from time to time on matters relating to health policy and services: regardless of the findings of this study, the outputs of this research would form part of that advice. ME: I have the following competing interests: I have received funding from the Academy of Finland for the study but the Academy had no involvement in its design, data collection, findings, or decision to publish.

## Authors’ contributions

HK contributed to the conception and design of the study, planning and performing of statistical and qualitative analyses, and drafted the manuscript. RL contributed to the conception and design of the study, performing the qualitative analyses and took part in the revision of the manuscript for important intellectual content. A-MA contributed to the conception and design of the study, planning and performing of statistical analyses, drafting the manuscript, and took part in the revision of the manuscript for important intellectual content. KM contributed to the conception and design of the study, performing the qualitative analyses, and took part in the revision of the manuscript for important intellectual content. IK contributed to the conception and design of the study, planning of analyses, and took part in the revision of the manuscript for important intellectual content. ME contributed to the conception and design of the study, planning of analyses, the collection of the data, and took part in the revision of the manuscript for important intellectual content. All authors have read and approved the final manuscript.
